# Colorimetric Immunoassay for Detection of Tumor Markers

**DOI:** 10.3390/ijms11125077

**Published:** 2010-12-07

**Authors:** Yongmei Yin, Ya Cao, Yuanyuan Xu, Genxi Li

**Affiliations:** 1 Department of Oncology, Jiangsu Province Hospital, Nanjing 210029, China; 2 Department of Biochemistry and National Key Laboratory of Pharmaceutical Biotechnology, Nanjing University, Nanjing 210093, China; E-Mails: cone_zimint@yeah.net (Y.C.); ladyxuyuanyuan@gmail.com (Y.X.); 3 Laboratory of Biosensing Technology, School of Life Sciences, Shanghai University, Shanghai 200444, China

**Keywords:** tumor markers, colorimetric immunoassay, nanomaterials

## Abstract

Tumor markers are substances, usually proteins, produced by the body in response to cancer growth, or by the cancer tissue itself. They can be detected in blood, urine, or tissue samples, and the discovery and detection of tumor markers may provide earlier diagnosis of cancer and improved therapeutic intervention. Colorimetric immunoassays for tumor marker detection have attracted considerable attention, due to their simplicity and high efficiency. The traditionally used colorimetric immunoassays for the detection of tumor markers are based on enzyme-linked immunosorbent assays, and the great achievement of nanotechnology has further opened opportunities for the development of such kind of immunoassays. This paper will summarize recent advances in the field of colorimetric immunoassays for detecting tumor markers, which is aimed to provide an overview in this field, as well as experimental guidance for the learner.

## 1. Introduction

Cancer is a leading cause of death worldwide, causing about 13% of all human deaths in 2007 (7.6 million), and deaths from cancer are projected to continue rising, with an estimated 12 million deaths projected for 2030. According to the World Health Organization, more than 30% of cancer deaths could be prevented if the cases were detected early and treated [1]. So, one key point of ultimate success for cancer treatment is the accurate diagnosis at the early stages.

Traditional methods for the diagnosis of cancer are endoscopy, biopsy and cytology specimen tests, as well as imaging/radiology tests, such as X-ray, positron emission computed tomography-computer tomography (PET-CT) and magnetic resonance imaging (MRI) [2–5]. Unfortunately, these diagnostic methods are not very powerful for the diagnosis of cancer at early stages, and some of these methods are quite time-consuming, expensive; thus not available for to a large number of people [6]. Therefore, the development of simple and rapid strategies that are specific and reliable for the diagnosis of cancer at early stages is of utter importance.

It has been known that cancer cells or other related non-tumor cells can release specific tumor markers, which are usually proteins, as tumors develop, into the circulation system in response to cancer growth. These tumor markers can be detected in blood, urine, or tissue samples, and the level of them is associated with the stage of cancer [7–10]. Alongside the development of proteomic technologies, lots of protein tumor markers have been discovered for many types of cancer (Table 1) [11–18]. Considering the high importance and potential application in the early diagnosis of cancer, detection of tumor markers has received more and more attention [19–23]. While some novel immunoassays have been developed [24–33], a variety of techniques have been employed for the development of these assays. Table 2 summarizes some examples of immunoassays which have been developed for tumor markers analysis [33–49]. Among the various detection techniques, the colorimetric method, in which the event is disclosed through a visual color change in the reaction medium, has proven the most convenient [50,51]. Thus, remarkable progress has been made on the design of colorimetric sensing systems for tumor marker detection over the years.

Large quantities of colorimetric assays for detecting tumor markers are proposed based on enzyme-linked immunosorbent assay (ELISA) [52–59]. Conventional ELISA-based colorimetric immunoassays may confront a serious sensitivity problem however, so low concentrations of tumor markers cannot be identified at the early stages of cancer. Therefore, research efforts have been focused mainly on the development of new signal amplification methods in colorimetric immunoassays. For instance, by incorporation of an enzyme-cascading step into the ELISA system, Lee *et al.* have reported a novel cascading ELISA, yielding detection limits of between 100 fM and 10 pM for both Prostate specific antigen (PSA) and Alpha-fetoprotein (AFP) in human serum [43]. Meanwhile, the emergence of nanotechnology is opening new horizons for highly sensitive detection of tumor markers [6,60–62]. Due to their excellent properties, many kinds of nanomaterials have been employed in colorimetric assays for detecting tumor markers [63–65].

Among the nanomaterials employed for the colorimetric detection of tumor markers, Au-NPs are the most commonly used, due to their unique optical properties. Their optical properties are strongly dependent on not only the size but also aggregation state of the particles, so smaller individual nanoparticles appear wine red while larger particles or aggregates of smaller particles range from purple to deep blue [45]. Besides, their excellent biocompatibility makes Au-NPs able to be fashioned with a wide range of biomacromolecules such as nuclear acids and enzyme-linked antibodies, greatly extending their application in the colorimetric detection of tumor markers. Therefore, large numbers of Au-NPs based colorimetric immunoassays have been developed for the detection of tumor markers, oncogenes and even tumor cells [66–70].

Apart from Au-NPs, magnetic particles (MPs) represent another exciting prospect in current analytical fields because they can be easily separated from a matrix by using a magnetic field [71–73]. Taking advantage of their unique magnetic characteristic as well as excellent biocompatibility, MPs have been widely utilized as a universal separation tool in the fabrication of colorimetric sensing systems for the detection of tumor markers [38,44,45,49,65,70].

## 2. Materials

### 2.1. ELISA-Based Colorimetric Immunoassays

#### 2.1.1. Chemicals

96-well microtiter plates and monoclonal/polyclonal capture antibodies are used for ELISA-based colorimetric immunoassays.

Enzyme-labeled secondary antibody: hydrogen peroxidase (HRP)-labeled and alkaline phosphatase (ALP)-labeled secondary antibody have been typically used in ELISA-based colorimetric immunoassays.

Enzyme substrates: The commonly used substrate for HRP is hydrogen peroxide (H_2_O_2_), coupled with several hydrogen donors, such as 3,3′,5,5′-tetramethylbenzidine (TMB: Supplier: Acros Organics N.V.; Catalog No. 229280010), *o*-phenylenediamine dihydrochloride (OPD: Supplier: Acros Organics N.V.; Catalog No. 218480250) and 2,2′-azino-di-[3-ethyl-benzothiazoline-6-sulfonic acid] diammonium salt (ABTS: Supplier: Roche Applied Science; Catalog No. 10102946001). The most widely used ALP substrate is p-nitrophenyl-phosphate (pNPP: Supplier: New England BioLabs; Catalog No. P0757S).

#### 2.1.2. Buffer Reagents

Carbonate/bicarbonate coating buffer: 100 mM phosphate buffered saline (PBS), containing 28.6 mM sodium carbonate (Na_2_CO_3_) and 71.4 mM sodium bicarbonate (NaHCO_3_), pH 9.6.

Washing buffer: 10 mM PBS, pH 7.4, containing 150 mM sodium chloride (NaCl) and 0.05% Tween-20.

Blocking buffer: PBS containing 0.1% bovine serum albumin (BSA) and 0.02% thimerosal.

All buffer reagents and other inorganic chemicals can be supplied by Sigma, Aldrich, or Fluka. All chemicals are used as received, and all aqueous solutions are prepared with doubly distilled water.

### 2.2. Nanomaterial-Based Colorimetric Immunoassays

Monodispersed Au-NPs with size from 2 to 250 nm can be commercially received from Ted Pella Inc. (Catalog No. 15701-15714). Au-NPs can be also synthesized in laboratory. Materials needed for the synthesis of Au-NPs are: hydrogen tetrachloroaurate (III) trihydrate (HAuCl_4_·3H_2_O, 99.9%. Supplier: Acros Organics N.V.; Catalog No. 411070010), trisodium citrate (Citrate·3Na. Supplier: Acros Organics N.V.; Catalog No. 391970025).

MPs or functionalized MPs can be also commercially received. For example, amino-functionalized superparamagnetic microparticles (size 1–2 μm) can be obtained from Polysciences, Inc. (Catalog No. 18879); streptavidin MagneSphere® paramagnetic particles (size 0.5–1.5μm) can be purchased from Promega Corporation. (Catalog No. MD1471).

## 3. Methods

### 3.1. ELISA-Based Colorimetric Immunoassays

The most frequently used ELISA method in colorimetric immunoassays for tumor marker detection is the two antibody “sandwich” ELISA, which measures the amount of antigen between two layers of antibodies [52]. In this assay, a capture antibody is firstly immobilized onto a solid support. The tumor marker, which is present in a biological sample or standard mixture, is then bounded and concentrated onto the support surface during incubation. After that, the solid support is incubated with a solution containing the detection antibody, thus the detection antibody will also bind with the tumor marker. Since the detection antibody is also tagged with certain enzymes, the color change caused by the enzyme-catalytic reaction can be thus used to quantify the amount of target tumor marker. The experimental details are described below, mainly with a slightly modified procedure recommended by Abcam [74].

#### 3.1.1. Coating with Capture Antibody

Coat the wells of a microtiter plate with the capture antibody at a concentration of 1–10 μg/mL in carbonate/bicarbonate coating buffer. Then cover the microtiter plate with an adhesive plastic. After incubation overnight at 4 °C, remove the coating solution and wash the plate at least twice by filling the wells with 200 μL of washing buffer. The solution in the wells can be removed by flicking the plate over a sink, and the remaining drops in the wells can be removed by patting the plate on a paper towel.

#### 3.1.2. Blocking and Adding Samples

200 μL of blocking buffer containing 5% non fat dry milk is firstly added into each well to block the remaining protein-binding sites. After that, the plate is covered with an adhesive plastic and incubated with the blocking buffer for at least 1–2 h at room temperature or overnight at 4 °C. Then add 100 μL of appropriately diluted samples into each well, and incubate the plate with the sample solution for 90 min at 37 °C. After incubation, remove the samples and wash the plate twice by filling the wells with 200 μL of washing buffer.

#### 3.1.3. Incubation with Detection Antibody

Dilute the detection antibody appropriately with 0.1 M bicarbonate buffer (pH 9.2). Then, add 100 μL of the buffer solution to each well. Cover the plate with an adhesive plastic and incubate for 2 h at room temperature. After that, wash the plate four times with washing buffer.

#### 3.1.4. Colorimetric Detection

Although many different types of enzymes have been used in ELISA, HRP and ALP are the two most widely used enzymes [43]. The substrate for HRP is H_2_O_2_, and the cleavage of H_2_O_2_ is usually coupled with the oxidation of a hydrogen donor which may change the color of the test solution during the reaction, so hydrogen donors should also be involved in the reaction. These hydrogen donors, usually TMB, OPD and ABTS, are light sensitive, so they need to be stored in the dark. Furthermore, OPD is considered hazardous (potential carcinogens), so it always needs to be handled with care. In a typical experiment, H_2_O_2_ and hydrogen donors are added into each well and incubated for 15–30 min. After that, equal volume of stopping solution (2 M H_2_SO_4_) is added to stop the reaction. Finally, the absorbance of the product of TMB oxidation is measured at the wavelength of 450 nm, while OPD at 492 nm; ABTS at 416 nm.

The most widely used ALP substrate is pNPP. The yellow color of the product nitrophenol can be measured at 405 nm after 15–30 min incubation at room temperature, and this reaction can be stopped by adding equal volume of 0.75 M NaOH.

### 3.2. Nanomaterial-based Colorimetric Immunoassays

The unique properties of nanomaterials may offer a wide range of opportunities in colorimetric immunoassays of tumor markers. In the following section, we provide experimental details for the synthesis, surface modification and utilization of nanomaterials in the fabrication of colorimetric sensing systems for the detection of tumor markers.

#### 3.2.1. Synthesis and Modification of Nanomaterials

##### 3.2.1.1. Synthesis of 13 nm Au-NPs

All glassware used in the synthesis procedures should be firstly immersed in freshly prepared aqua regia (HNO_3_: HCl = 1:3) for 30 min, then washed with water and dried before use. Au-NPs are synthesized by reducing HAuCl_4_ with Citrate·3Na. Briefly, a 100 mL aqueous solution of 0.01% (w/v) HAuCl_4_ is added into a round-bottom flask and stirred to boil. Then, 3.5 mL 1% trisodium citrate is added rapidly into the boiling solution, the color of which changes from colorless to wine red after boiling for another 15 min with vigorous stirring. The size of the nanoparticles is 12.5 ±2.3 nm, which can be determined by transmission electron microscope (TEM). The concentration of Au-NPs is 3.5 nM, which can be calculated from the quantity of starting material (HAuCl_4_) and the size of Au-NPs at a wavelength of 520 nm [45].

##### 3.2.1.2. Modification of Au-NPs

The main technique reported for the modification of Au-NPs surfaces is based on Au-S covalent bond formation between the modified molecules and the gold atoms on the particle surface [71], which makes use of sulphur-containing ligands, such as thiol, disulphide and thiolester.

###### 3.2.1.2.1. Modification of Au-NPs with Thiol-oligonucleotides

The above prepared Au-NPs solution is firstly condensed by centrifugation at 12,000 rpm for 20 min at 4 °C to a 1 mL Au-NPs solution, while 800 μL supernatant is removed; the remaining 200 μL is redispersed. After condensation, 800 μL Au-NPs solution is mixed with 200 μL, 5 μM 5′-thiol-oligonucleotides at room temperature. After 24 h duration in darkness, the mixture is centrifuged twice, each for 20 min at 12,000 rpm to remove the excess thiol-oligonucleotides. After centrifugation, the precipitate is washed with 4 mM Citrate·3Na. The Au-NPs, modified with thiol-oligonucleotides, are stored at 4 °C when not in use. To determine the number of thiol-oligonucleotides modified on each Au-NP, 2-mercaptoethanol (1.0 M, 10 μL), which has a smaller size than oligonucleotides and is easier to be modified on the surface of Au-NPs through Au-S bond, is used to replace thiol-oligonucleotides from the surface of Au-NPs (11 nM, 990 μL). The amount of replaced thiol-oligonucleotides in the supernatant liquor can be thus used to calculate the quantity on each Au-NP [75].

###### 3.2.1.2.2. Modification of Au-NPs with Antibodies

The methods to modify Au-NPs with antibody include adsorption [76], *N*-hydroxysuccinimide (NHS) ester chemistry [77], and oligonucleotide-directed immobilization [78]. Among them, the direct adsorption method is the simplest one, due to the fact that neutral or weakly negative-charged proteins can be adsorbed on the surface of Au-NPs easily and stably through coordinate-covalent bond [79]. The preparation of Au-NPs modified with antibodies through direct adsorption is as follows [45]: 20 μL of protein solution (1 mg/mL) is first added into 5 mL of the above prepared Au-NPs solution, and the pH is appropriately adjusted by adding 1 M NaOH solution. Then the mixed solution is incubated for 30 min at 37 °C with gentle shaking, and centrifuged to 1 mL (12,000–13,000 rpm, 20–30 min, 4 °C). After that, 100 μL of 10% (w/v) BSA solution, used as non-specific blocker, is added into the solution to passivate the surface of Au-NPs. After 30 min, the mixed solution is again centrifuged (12,000–13,000 rpm, 20–30 min, 4 °C). The supernatant is subsequently decanted, and the precipitate is rinsed with 1 mL of PBS buffer. The centrifuging/rinsing procedure should be repeated three times to remove the unbounded antibodies and BSA.

##### 3.2.1.3. Modification of MPs

MPs may provide an efficient tool of separating target analytes from the liquid suspension; however, unmodified MPs have some limitations in the applications. Therefore, MPs surfaces should first of all be modified with some specific linking groups for further biomolecules binding, including: (1) Active chemical groups (e.g., carboxyl and amino groups), which can covalently bind biomolecules in the presence of specific cross-linking reagents; (2) streptavidin/avidin ligands, which can be specifically attached to biotinylated biomolecules [71–73]. Taking into account that amino- or streptavidin-functionalized MPs are commercially available, the following is only a description of the modification by antibodies for these pre-functionalized MPs.

###### 3.2.1.3.1. Modification of Amino-Functionalized MPs

Amino-functionalized MPs can be modified with antibodies by using glutaraldehyde-amine coupling chemistry [33]. Briefly, amino-functionalized MPs (0.05 mM) in 1 mg mL^−1^ of ethylenediaminetetraacetic acid (EDTA) solution (5 mL) are firstly washed with 5% (v/v) pyridine washing buffer (10 mL). Then, MPs are separated magnetically. This process should be repeated three times. After that, the MPs are activated by glutaraldehyde in pyridine washing buffer (5 mL, 5%) for 3 h at room temperature, separated magnetically twice, and resuspended in pyridine washing buffer (10 mL). Finally, antibody dissolved in pyridine wash buffer (1 mL at 1 mg mL^−1^) is added to the MPs solution. After 10 h duration at room temperature, 1 mg BSA is added to the MPs solution. After another 10 h at room temperature, the MPs are washed twice and then resuspended in pyridine washing buffer (5 mL). Subsequently, glycine solution (3 mL, 1 M at pH 8.0) is added into the above solution to quench all of the unreacted aldehyde groups, and the resulting solution is stirred for 30 min. After magnetic separation three times and the washing step, MPs modified with antibody (Ab-MPs) are resuspended in PBS solution.

###### 3.2.1.3.2. Modification of Streptavidin-Functionalized MPs

Streptavidin-functionalized MPs are firstly washed three times with washing buffer (75 mM NaCl, 7.5 mM Citrate·3Na, pH 7.0) prior to use. Then they are diluted to 0.1 mg mL^−1^ with PBS, pH 7.4. After that, a solution of biotinylated antibody is added to the MPs solution at a ratio of 100 μg protein to 1 mg MPs, and the mixed solution is incubated for 20 min at room temperature. Finally, the Ab-MPs are thoroughly washed three times with pH 7.4 PBS [45].

#### 3.2.2. Magnetic Collection and Separation of Target Tumor Markers

MPs modified with capture antibodies can be used for fast target protein collection and separation. In a typical experiment, 50 μL of capture antibody-modified MPs (0.2 mg mL^−1^) are first added to a 200 μL eppendorf tube. Then, 50 μL of assay buffer containing various concentrations of target tumor markers are added. After incubation for 30 min at 37 °C with gentle shaking, the magnetic beads are magnetically collected and twice rinsed with washing buffer [70].

#### 3.2.3. Application of Au-NPs in Colorimetric Immunoassays of Tumor Markers

Basically, two colorimetric procedures by making use of Au-NPs have been proposed for tumor marker quantification: (a) Analysis of the state change of Au-NPs in solution, including the homogenous growth and aggregation triggered by a biological process; (b) analysis of the activity of enzymes that attached to Au-NPs, in which case Au-NPs act as enzyme carriers and signal enhancers.

##### 3.2.3.1. Colorimetric Assay Using Homogenous Growth of Au-NPs

10 mL solution for the growth of Au-NPs is first prepared in a 20 mL vial, consisting of final effective concentrations of 0.25 mM HAuCl_4_ and 0.1 M cetyltrimethylammonium bromide (CTAB). Before use, the solution is gently mixed with ascorbic acid (AA) solution with a final AA concentration of 10 mM and stirred. Seperately, Au-NPs/tumor marker complexes are first separated through magnetic separation and washed with 0.01 M PBS buffer (pH 7.4) three times to remove the excess of free Au-NPs. Then, Au-NPs are dissociated from the complexes by using 50 mM NaCl/1 M NaOH elution solution. Finally, the upper aqueous solution containing the dissociated Au-NPs is transferred to a 1 mL cuvette, followed by addition of the above previously prepared 1 ml solution for the growth of Au-NPs. The growth of Au-NPs and the color change of the solution are then recorded [38].

##### 3.2.3.2. Colorimetric Assay Using Aggregation of Au-NPs

In this section, we introduce the experimental details of two typical assays. One is based on single chain fragment variable recombinant antibody (scFv)-functionalized Au-NPs, while the other is colorimetric bio-barcode assay.

###### 3.2.3.2.1. scFv-Functionalized Au-NPs Based Assay

Firstly, scFv-functionalized Au-NPs are prepared as described in Section 3.2.1.2.2. Then, a range of concentrations of target protein are added to the solution of the scFv-functionalized Au-NPs while stirring, thus the scFv-functionalized Au-NPs show visible color change due to the aggregation of the nanoparticles caused by the interaction of scFv with the target protein. After 30 min, the developing reaction can be recorded by using a UV-visible spectrophotometer [69].

###### 3.2.3.2.2. Colorimetric Bio-Barcode Assay

In a typical experiment, two types of probes are prepared. The first is a magnetic probe, *i.e.*, MP modified with monoclonal antibodies for target tumor marker, prepared as described in Section 3.2.1.3.1. The second is a barcode probe, *i.e.*, a porous silica particle modified with the monoclonal antibodies and barcode DNA complements. To prepare the barcode probe, amino-functionalized porous silica microparticles are firstly linked to antibodies using glutaraldehyde-amine coupling chemistry. Then, 3′-amino-functionalized barcode DNA complements (1 mL at 100 μM) are added to the solution containing the antibody modified silica particles. After incubation overnight at room temperature with gentle shaking, the solution is centrifuged twice, and the pellets are resuspended in 1 mL of 0.2 M ethanolamine. After 30 min duration at room temperature, centrifugation is performed again to remove the supernatant, and a 10% BSA solution is subsequently added, followed by repeating the centrifugation steps twice. Finally, the resulting pellets are resuspended in 1 mL of 0.15 M PBS solution, which is further mixed with 30 μL 100 μM barcode DNA solution and incubated for 1 h at room temperature.

In the tumor marker detection assay, 15 μL of magnetic probe solution (1.5 × 10^9^ beads/mL) is first added to 20 μL of tumor marker solution, followed by the addition of 15 μL of barcode probe solution (1 × 10^9^ beads/mL). The mixed solution is incubated at 37 °C for 50 min on an orbital shaker. Then, the solution is magnetically separated as described in Section 3.2.2, and the magnetically separated complexes are washed with 0.15 M PBS solution three times. After that, 50 µL of NANOpure water (18 MΩ) is added to the complexes, and the solution is kept on a rocking shaker at 70 °C for 10 min to release the barcode DNA. Finally, the supernatant containing free barcode DNA strands is collected for barcode DNA detection. To detect the barcode DNA, DNA-functionalized Au-NPs (prepared as described in Section 3.2.1.2.1) are added to the barcode DNA in 0.15 M PBS solution. Due to the hybridization between the barcode DNA and its complements that are modified on Au-NPs surfaces, Au-NPs aggregate and show visible color change. And, the mixed solution, after being maintained at room temperature for 2 h, can be transferred into a 100-μL quartz cuvette to measure the extinction coefficients by using a UV-vis spectrophotometer [29,68].

##### 3.2.3.3. Colorimetric Assay using Enzymes Labeled Au-NPs

Due to the high loading of signaling molecules on each nanoparticle, greatly enhanced sensitivity for the detection of tumor markers can be achieved by using enzyme labeled Au-NPs [70]. In such colorimetric assays, Au-NPs play a role of enzyme carriers and signal amplifiers, and the detectable signals come from the catalytic reaction of the enzymes that are loaded onto the nanoparticle surfaces. The commonly used enzymes and substrates are the same as those used in ELISA-based colorimetric assays.

## 4. Results and Discussion

**Figure 1.**
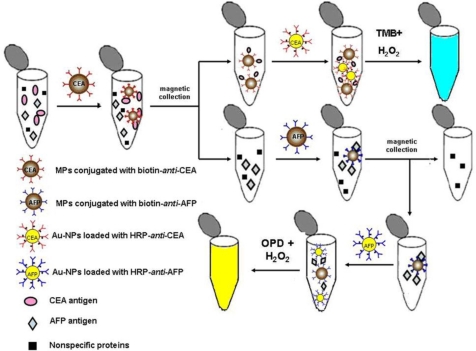
Scheme of the colorimetric multiplexed immunoassay for sequential detection of tumor markers, CEA and AFP. Reprinted with permission from Ref. [45]. Copyright 2009 Elsevier.

As an example, one recent work in the authors’ laboratory on colorimetric multiplexed immunoassays is introduced [45]. Figure 1 illustrates the principle of the assay for sequential detection of tumor markers. Briefly, biotin-anti-CEA conjugated MPs are first added to the test solution that contains CEA, AFP and some nonspecific proteins. After magnetic separation, CEA antigen which is captured by biotin-anti-CEA, immobilized on the surface of MPs, is thus collected. Therefore, if Au-NPs loaded with HRP-anti-CEA are added to the collected CEA antigen solution, HRP-anti-CEA loaded on the surface of Au-NPs may capture the CEA antigen conjugated on the surface of MPs. Consequently, when TMB and H_2_O_2_ are added to the test solution, its color turns blue. Alternatively, the detection of AFP antigen in the test solution can be carried out by repeating the above procedure, using biotin-anti-AFP conjugated MPs and Au-NPs loaded with HRP-anti-AFP.

**Figure 2.**
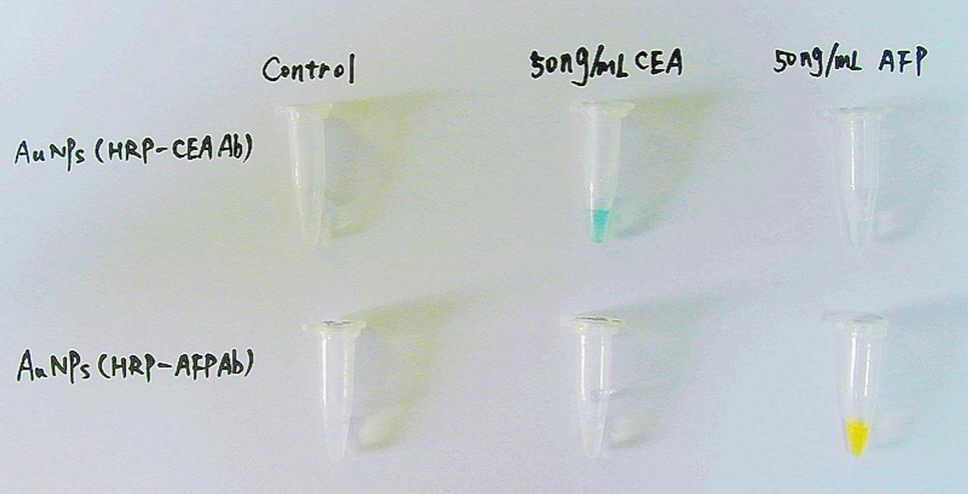
Colorimetric detection of CEA and AFP antigens. Only in the presence of antigens which are cognate with the antibodies loaded on the surfaces of MPs and Au-NPs, can the immunocomplexes be formed, and the characteristic blue and yellow colors of catalytic products be observed. Reprinted with permission from Ref. [45]. Copyright 2009 Elsevier.

The multiplexed immunoassay is very simple and easily operated, and the results can be seen by the naked eye (Figure 2). Meanwhile, the absorbance at 370 nm or 490 nm of the test solutions can be separately used to determine the amount of tumor markers more accurately. Under optical conditions, the immunoassay can provide sequential detection of CEA and AFP with detection limits of 0.02 ng/mL (≈0.1 pM), which can be satisfactory for clinical applications.

Colorimetric assay is very simple and easily operated, without requiring the expensive instruments needed in the optical immunoassay systems, such as charge-coupled device (CCD) camera and multi-channel injection valves fixed to luminescence analyzers. In fact, electrochemical assay is also very simple and relatively inexpensive, thus some electrochemical methods have also been proposed for the detection of cancer. However, the current amplification strategy for electrochemical signal often involves multiple steps of deposition and stripping, making the experimental process complex; while colorimetric approaches do not require complicated experimental steps, and results can be seen with the human naked eye. Therefore, colorimetric immunoassay for the detection of tumor markers has received more and more interest. Meanwhile, nanotechnology has greatly promoted the development of colorimetric immunoassays. Firstly, MPs may provide a rapid and safe approach to separate the target from the other species, preventing the interference of non-specific proteins in immunoassays. Secondly, the unique optical properties of Au-NPs can be harnessed to realize the transduction of the presence of target tumor markers to easily detectable signals, simplifying the designed detection system. Thirdly, the use of nanomaterials has enabled detection of tumor markers with greater sensitivity and accuracy. Therefore, colorimetric immunoassays have made prominent progress in the detection of tumor markers and may play an important role in the early diagnosis of cancer.

Colorimetric immunoassays have many desirable merits, thus are rapidly developing. Tremendous opportunities and challenges exist in the application of colorimetric methods for tumor marker detection.

Firstly, colorimetric immunoassays for tumor marker detection are usually proposed by using antibodies; however the availability of antibodies with high affinity and low cross-reactivity are very limited. Fortunately, aptamers—nucleic acid molecules that can selectively bind to low molecular weight organic or inorganic substrates or to macromolecules such as proteins—can be considered as attractive alternatives to antibodies [45]. In fact, compared with antibodies, aptamers may possess many advantages, such as relatively simple and inexpensive synthesis, tolerance to internal labeling, high stability, and stronger and more selective affinity for protein targets. Therefore, several aptamers have been employed for tumor marker detection [80,81].

Secondly, nanomaterials used in colorimetric methods for sensing tumor markers are still narrowly restricted to Au-NPs and MPs. Recently, some novel types of nanomaterials were fabricated and used in colorimetric biosensors for various analytes [82–84], so more nanomaterials will be applied in the development of colorimetric immunoassays for tumor marker detection, simplifying the assay methodology and lowering the detection limit as far as possible.

Finally, the measurement of a single tumor marker is usually not sufficient for diagnosis purposes, because most cancers have more than one marker associated with their incidence [85], thus multiplexed immunoassays that can quantitatively measure the concentrations of multiple tumor markers in a single assay have received more and more interest [25,46]. Compared with the traditional parallel single-analyte immunoassay, multiplexed immunoassays have the advantage of simplified analytical procedure, shortened analysis time, decreased sample consumption, increased test throughput, reduced costs, and improved diagnostic accuracy [45]. Consequently, future advances in colorimetric immunoassays will depend on the development of simple and efficient sensing mechanisms for multiplexed tumor marker examinations.

## Figures and Tables

**Table 1.** Some tumor markers currently in use.

**Tumor Markers**	**Related Cancers**	**Usual Sample**	**Refs.**
AFP (Alpha-fetoprotein)	Liver, germ cell cancer of ovaries or testes	Blood	[11]
CA 15-3 (Cancer antigen 15-3)	Breast	Blood	[7]
CA-125 (Cancer antigen 125)	Ovarian	Blood	[12]
CEA (Carcinoembryonic antigen)	Colorectal, breast, thyroid *et al.*	Blood	[13]
Estrogen receptors	Breast	Tissue	[14]
hCG (Human chorionic gonadotropin)	Testicular and trophoblastic disease	Blood, urine	[15]
Her-2/neu	Breast	Tissue	[16]
Progesterone receptors	Breast	Tissue	[14]
PSA (Prostate specific antigen)	Prostate	Blood	[17,18]

**Table 2.** Examples of immunoassays for tumor markers analysis.

**Tumor markers**	**Biosensor principle**	**Assay principle**	**Limit of detection**	**Refs.**
PSA	Fluorescence	Fluorophore-based bio-barcode amplification method	30 nM	[33]
PSA	Microcantilever	Microcantilever	2 nM	[34]
PSA	Electrochemistry	Using gold nanoparticle film electrodes and multienzyme-particle amplification	5 fM	[35]
PSA	Electrochemistry	Carbon nanotube amplification strategies	40 fM	[36]
PSA	Surface-Enhanced Raman Scattering	Immunoassay based on Surface-Enhanced Raman Scattering and immunogold labels	30 fM	[37]
PSA	Colorimetry	Homogenous growth of gold nanocrystals	10 fM	[38]
AFP	Fluorescence	Fluorescence quenching signal of gold nanoparticles	0.17 nM	[39]
AFP	Mass spectrometry	Mass spectrometry signal amplification using small-molecule tagged gold microparticles	1 nM	[40]
AFP	Electrochemistry	Amperometric enzyme immunosensor based on gold nanoparticles and multi-walled carbon nanotube composite membranes	0.6 pM	[41]
AFP	Chemiluminescence	Multilayers enzyme-coated carbon nanotubes as label	0.1 pM	[42]
AFP	Colorimetry	Cascade enzyme-linked immunosorbent assay	0.1 pM	[43]
AFP	Colorimetry	DNAzyme functionalized nano-probes	1.4 pM	[44]
CEA, AFP	Colorimetry	Colorimetric multiplexed immunoassay based on gold nanoparticles	0.02 ng/mL; 0.1 pM	[45]
CEA	Chemiluminescence	Flow-through multianalyte system with substrate zone-resolved technique	0.6 ng/mL	[46]
CEA	Electrochemistry	Layer-by-layer assembly of gold nanoparticles-multi-walled carbon nanotubes-thionine multilayer films	0.01 ng/mL	[47]
CEA	Surface-Enhanced Raman Scattering	Surface-Enhanced Raman Scattering of hollow gold nanospheres	0.01 ng/mL	[48]
CEA	Colorimetry	Enzyme-labeled gold nanoparticle probes	0.012 ng/mL	[49]
